# Supplementary material for: DCAF15 control of cohesin dynamics sustains acute myeloid leukemia
Source: Nat Commun. 2024 Jul 3;15:5604. doi: 10.1038/s41467-024-49882-x (PMC11222469; doi:10.1038/s41467-024-49882-x)
Supplement: Supplementary file 1 — Supplementary Information [file 41467_2024_49882_MOESM1_ESM.pdf]

DCAF15 control of cohesin dynamics sustains acute myeloid leukemia

Supplementary Information

Supplementary Methods

Non-competitive cell proliferation assay
Cas9+ MV4-11 and OCI-AML3 cell lines were transduced with LRG2.1 sgRNA-containing lentiviral vector co-expressed with a GFP reporter. On day 3 post-infection, GFP+ cells were sorted to obtain pure sgRNA-transduced populations. On day 4 post-infection, cells were plated in 24-well plates at 10,000cells/ml. Every 3-4 days thereafter until day 21, total cell count was determined by flow cytometric analysis, and cells were re-plated at the original starting density of 10,000cells/ml. Cumulative number of cells was used for analysis.

siRNA transfection
HEK293T cells seeded at ~50% confluency in 10-cm plates were transfected with 600pmol siRNA and 30µL Lipofectamine-2000 (Thermo Fisher Scientific, 11668027) for 48h. Cells were incubated with siRNA transfection reagents for 6h prior to removing and refreshing media. The following siRNAs were used: ON-TARGETplus Human RAD21 (5885) siRNA SMARTPool (Horizon Discovery, L-006832-00-0005).

Cycloheximide-chase assay
$5x10^6$ MV4-11 cells stably expressing a doxycycline-inducible shRNA targeting *DCAF15* were plated in 10-cm plates and treated with DMSO or doxycycline (Millipore Sigma, D9891) for 48h, and then 20µg/ml cycloheximide for 0, 0.5, 1, 2, and 4h, respectively, prior to immunoblotting.

Cell cycle analysis
Cells were stained with 10µM Hoechst 33342 solution (Thermo Scientific, 62249) for 30min, and then subjected to flow cytometric analysis for cell cycle phase quantification.

In vitro translation (IVT)
In vitro translation (IVT) was performed using a kit (TNT Coupled Reticulocyte Lysate Systems, Promega, L1170) that contains reaction buffer, rabbit reticulocyte lysate, RNA polymerase, 1mM methionine, and 1mM amino acid mixture (minus methionine). Reaction components were assembled on ice according to the manufacturer's protocol, HA-tagged *DCAF15* cDNA plasmid was used as a DNA template, and nuclease free water was added to a final volume of 50µl. Reaction mixture was incubated at 30°C for 90min, and translated protein was subjected to SDS-PAGE and analyzed by immunoblot.

Immunofluorescence staining and confocal microscopy
Cells (400,000/well) were seeded into a 24-well plate containing cover glasses pre-treated with poly-D-lysine and centrifuged at 796 x g for 10min. Subsequently, the cells were fixed with 4% PFA in PBS for 15min, washed 3x with PBS (5 min each time), permeabilized with PBS containing 0.1% Triton-X 100 for 10 minutes, and washed again with PBS 3x (5min each time). Following this, cells were blocked in PBS with 10% goat serum for 30min and then incubated overnight with primary antibodies. After 3 washes in PBS (5min each time), cells were incubated with secondary antibodies for 1h in the dark. Further washing with PBS was done 3x (5min each time), followed by mounting with DAPI-containing mounting media. The primary antibody, γH2AX (Millipore-Sigma, 05-636), was diluted 1:200 in PBS with 1% BSA and 0.1% Tween-20, while the secondary antibody, goat anti-Mouse IgG (H+L) AlexaFluor 568 (Invitrogen, A11031), was diluted 1:250 in PBS with 1% BSA and 0.1% Tween-20. Images were captured using an

inverted laser scanning confocal microscope (Zeiss, LSM880) with a 63x oil DIC objective under the control of the Zeiss Zen software.

ChIP-qPCR

$2x10^7$ cells were collected, washed, and cross-linked with 1% paraformaldehyde in PBS for 10min and quenched with 125mM glycine for 5 min at room temperature. Fixed cells were suspended in lysis buffer (0.1% SDS, 1% Triton X-100, 10mM Tris-HCl, 1mM EDTA, 0.1% NaDOC, 300mM NaCl, 0.25% sarkosyl, 1mM DTT, and protease inhibitors) and sonicated in Covaris Ultrasonicator. Samples were pre-cleared with 10μl of BSA-coated Protein A Dynabeads (Thermo Fisher, 10001D) at 4°C for 1h and incubated with 20μg of antibody overnight. The following antibody for ChIP-qPCR was used: α-ESCO1 (gift from Susannah Rankin, Oklahoma Medical Research Foundation, 20μg per IP). The antibody-conjugated samples were incubated with 75μl of BSA-coated Protein A Dynabeads at 4°C for 1h. The immunoprecipitants were washed twice with low-salt buffer (150mM NaCl, 0.1% SDS, 1% Triton X-100, 1mM EDTA, and 50mM Tris-HCl), twice with high-salt buffer (500mM NaCl, 0.1% SDS, 1% Triton X-100, 1mM EDTA, and 50mM Tris-HCl), twice with LiCl buffer (150mM LiCl, 0.5% Na-Deocycholate, 0.1% SDS, 1% NP-40, 1mM EDTA, and 50mM Tris-HCl), and once with TE buffer (1mM EDTA, 10mM Tris-HCl). Bound DNA was eluted with 200μl of elution buffer (1% SDS, 200mM NaCl, 10mM EDTA, and 50mM Tris-HCl) at 65°C overnight. Eluted DNA was incubated with 2μl of 0.5mg/ml RNase A (Fisher Scientific, BP2539250) at 37°C for 1h, incubated with 2μl of 20mg/ml Proteinase K (Thermo Fisher, EO0491) at 55°C for 1h, and purified with QIAquick PCR purification kit (Qiagen, 28104).

For ChIP-qPCR, ChIP DNA was diluted 100-fold using nuclease-free water for quantitative real-time PCR analysis with the ViiA7 Real-time PCR System (Thermo Fisher) and the Power SYBR Green PCR Master Mix (Fisher-Scientific, A25778). ChIP-qPCR primer sequences (P1-P5) are from Rahman *et al.*[1] and are listed below:

P1: CTTCCTGGGCTCAAGATGTC (forward), TTTTCAGGGATAGCCTGGTG (reverse)
P2: AAGCAAAGTCCTCCGTTCAG (forward), TATGGCAGATGCCTAACAGG (reverse)
P3: GTTTCAGCTTGCCATCAACC (forward), TGCACCAGCCTTCACTTATC (reverse)
P4: ACAGGCCCGTGTTTATTCTG (forward), CATGGCTCCTCAAGTCACTC (reverse)
P5: GGGCACAAATGACTTTCCAG (forward), CATGTCATGGTAGAGCCAAGC (reverse)

Supplementary Table 1. Antibodies used for Western blotting.

Target Name	Manufacturer	Catalog #	Dilution
Primary Antibodies			
CAPER ("RBM39")	Bethyl Laboratories	A300-291A-T	1:2,000
HA-Tag	Cell Signaling Technology	3724S	1:2,000
Vinculin	Santa Cruz Biotechnology	sc-73614	1:8,000
p21	Santa Cruz Biotechnology	sc-6246	1:250
p53	Bethyl Laboratories	A300-247A-T	1:1,000
Cleaved Caspase-3 (Asp175)	Cell Signaling Technology	9661T	1:500
SMC1 ("SMC1A")	Bethyl Laboratories	A300-055A-T	1:1,000
SMC3	Bethyl Laboratories	A300-060A-T	1:1,000
RAD21	Bethyl Laboratories	A300-080A-T	1:1,000
SA2 ("STAG2")	Bethyl Laboratories	A302-580A-T	1:1,000
SCC-112 ("PDS5A")	Bethyl Laboratories	A300-089A-T	1:1,000
CDCA5 ("Sororin")	Abcam	ab192237	1:500
Cyclin D2	Cell Signaling Technology	3741T	1:1,000
DDB1	Bethyl Laboratories	A300-462A-T	1:1,000
FLAG-Tag	Millipore Sigma	F7425	1:6,000
WAPL	Proteintech	16370-1-AP	1:500
SA1 ("STAG1")	Bethyl Laboratories	A302-579A-T	1:2,000
PDS5B	Bethyl Laboratories	A300-538A-T	1:2,000
Ubiquitin (K48)	Cell Signaling Technology	8081S	1:1,000
Acetyl-SMC3 (Lys105/106)	Millipore Sigma	MABE1073	1:1,000
HDAC8	Proteintech	17548-1-AP	1:500
Phospho-Cdc2 (Tyr15)	Santa Cruz Biotechnology	sc-136014	1:250
Cdc25A	Santa Cruz Biotechnology	sc-7389	1:250
Phospho-Histone-H3 (Ser10)	Millipore Sigma	06-570	1:1,000
Gamma-H2AX	Bethyl Laboratories	A300-081A-T	1:500
VprBP	Bethyl Laboratories	A301-887A	1:1,000
MCM4	Proteintech	13043-1-AP	1:1,000
Cyclin E1	Cell Signaling Technology	4129T	1:1,000
Secondary Antibodies			
Goat anti-rabbit IgG, HRP-linked	Cell Signaling Technology	7074S	1:10,000
Sheep anti-mouse IgG, HRP-linked	Cytiva	NA931-1ML	1:10,000

Supplementary References

1. Rahman, S., K Jones, M. J. & Jallepalli, P. V. Cohesin recruits the Esco1 acetyltransferase genome wide to repress transcription and promote cohesion in somatic cells. *PNAS* **112**, 11270–11275 (2015).
2. Barretina, J. *et al.* The Cancer Cell Line Encyclopedia enables predictive modelling of anticancer drug sensitivity. *Nature* **483**, (2012).
3. Tang, Z. *et al.* GEPIA: a web server for cancer and normal gene expression profiling and interactive analyses. *Nucleic Acids Res* **45**, (2017).
4. Karpinich, N. O., Tafani, M., Rothman, R. J., Russo, M. A. & Farber, J. L. The Course of Etoposide-induced Apoptosis from Damage to DNA and p53 Activation to Mitochondrial Release of Cytochrome c. *Journal of Biological Chemistry* **277**, 16547–16552 (2002).
5. Jumper, J. *et al.* Highly accurate protein structure prediction with AlphaFold. *Nature* **596**, 583 (2021).
6. Goddard, T. D. *et al.* UCSF ChimeraX: Meeting modern challenges in visualization and analysis. *Protein Science* **27**, 14–25 (2018).
7. Pettersen, E. F. *et al.* UCSF ChimeraX: Structure visualization for researchers, educators, and developers. *Protein Science* **30**, 70–82 (2021).
8. Nishiyama, T. *et al.* Sororin Mediates Sister Chromatid Cohesion by Antagonizing Wapl. *Cell* **143**, 737–749 (2010).
9. Zhang, Y. *et al.* Model-based analysis of ChIP-Seq (MACS). *Genome Biol* **9**, 1–9 (2008).
10. Machanick, P. & Bailey, T. L. MEME-ChIP: motif analysis of large DNA datasets. *Bioinformatics* **27**, 1696–1697 (2011).

Supplementary Figure 1. *DCAF15* is an AML-biased E3 ubiquitin ligase dependency.

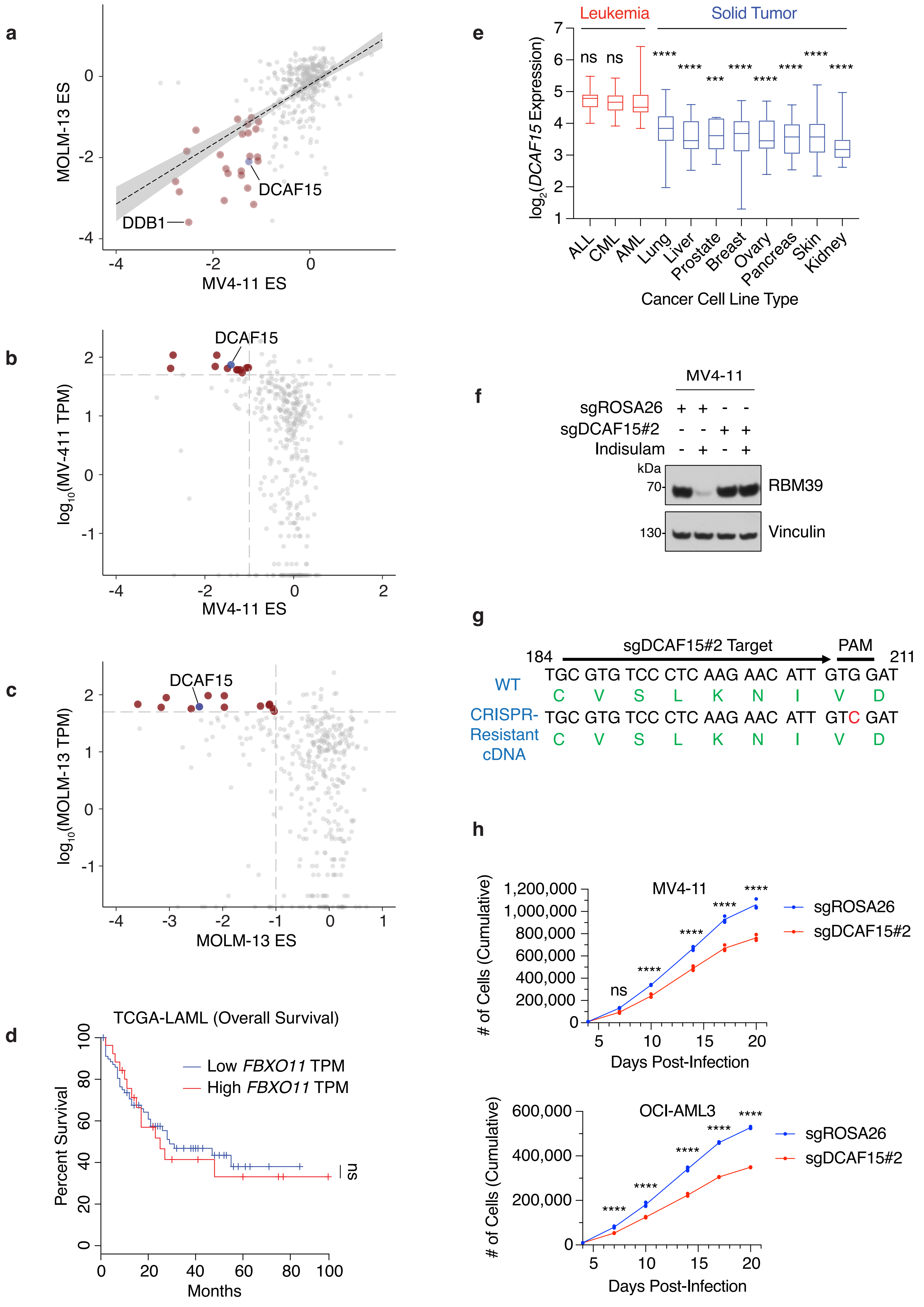

Supplementary Figure 1. *DCAF15* is an AML-biased E3 ubiquitin ligase dependency.

- **a.** Scatter plot showing the correlation between MV4-11 ES and MOLM-13 ES for individual genes as determined in the CRL domain-focused CRISPR dropout screens.
- **b**. Scatter plot showing the relationship of mRNA expression and CRISPR ES. Dropout hits (ES<-1) in MV4-11 cells are shown in red. Gene mRNA expression values were extracted from Broad Institute Cancer Cell Line Encyclopedia (CCLE)[2].
- **c.** Same as in (b), except that MOLM-13 cells were utilized.
- **d.** Overall survival of AML patients using GEPIA platform[3] with low (bottom 75%) or high (top 25%) *FBXO11* mRNA expression (TCGA-LAML dataset [https://www.cancer.gov/tcga]). TPM, Transcripts Per Million. n=106. log-rank test, ns=not-significant (p=0.78).
- **e.** Box plots showing *DCAF15* mRNA expression across various cancer cell line types analyzed from CCLE RNA-seq data. Welch's two-sided t-tests of AML mean versus other means, ****=p-value<0.0001; ***=p-value<0.001 (p=0.0006); ns=not-significant. Center line shows median, box limits show 75th and 25th percentiles, whiskers show minimum-maximum values.
- **f.** MV4-11 Cas9+ cells stably expressing lentiviruses encoding *ROSA2*6 (negative control)- or *DCAF15*-targeting sgRNAs were treated with DMSO or 3μM indisulam for 6h. Lysates were analyzed by Western blot for the indicated proteins. Immunoblots are representative of three independent experiments.
- **g.** Schematic describing the design of a CRISPR-resistant *DCAF15* cDNA. Encoded amino acids are labeled in green font beneath the cDNA sequences. PAM-disrupting nucleotide substitution (nucleotide [nt]-G207C) is indicated in red font.
- **h.** Non-competitive proliferation assay performed in Cas9+ MV4-11 (Top) and OCI-AML3 (Bottom) cell lines infected with lentiviruses containing *ROSA26*-targeting (negative control) or *DCAF15*-targeting sgRNAs and sorted to obtain pure populations. Cells were counted every 3-4 days and replated at original density of 10,000 cells/ml. Cumulative number of cells was calculated over 21 days. n=3 biologically independent replicates. Two-way ANOVA (with Bonferroni's multiple comparisons test), ****=padj<0.0001, ns=not-significant.
- Source data are provided as a Source Data file.

Supplementary Figure 2. *DCAF15* loss suppresses AML via activation of p53.

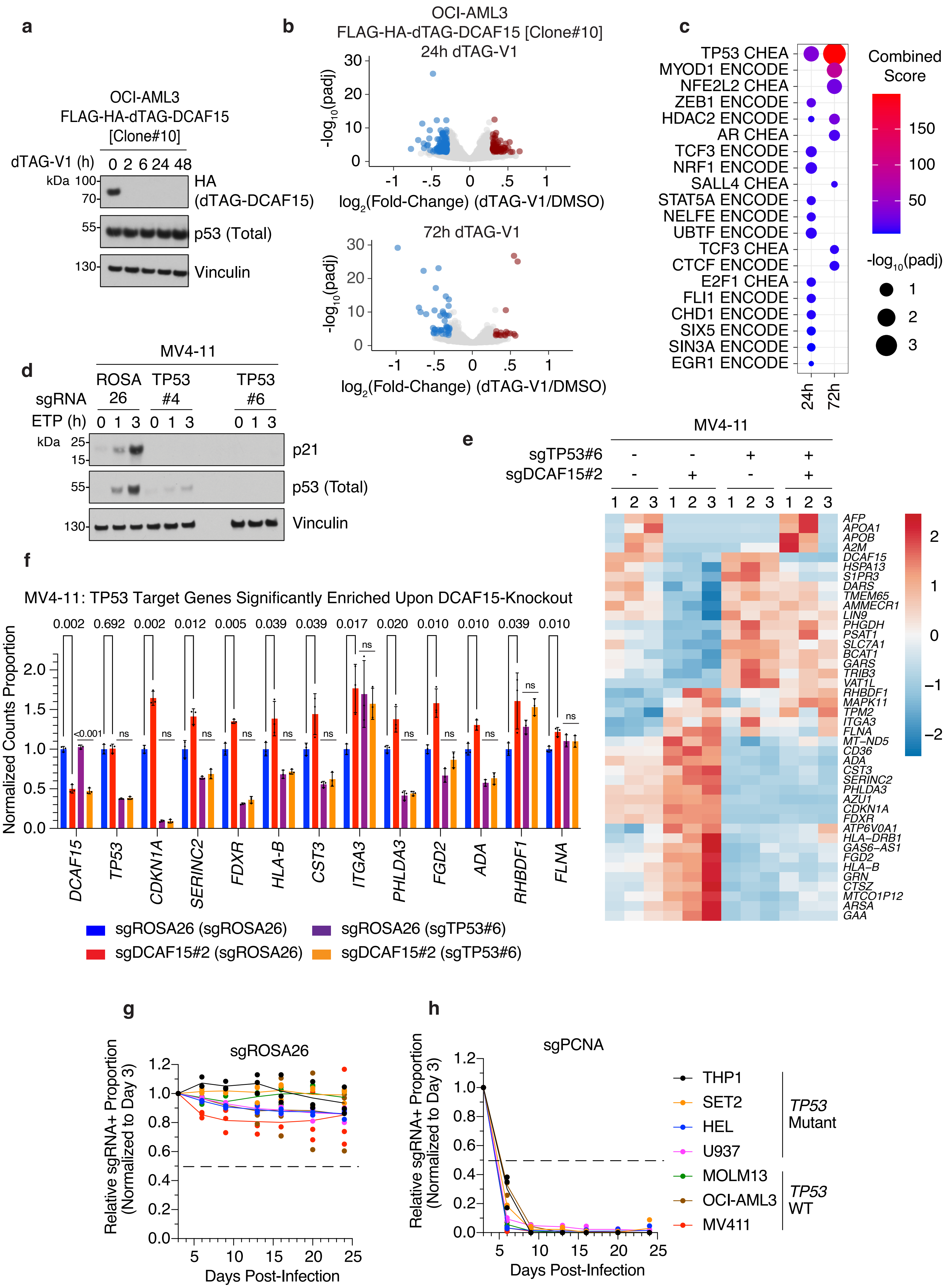

Supplementary Figure 2. *DCAF15* loss suppresses AML via activation of p53.

- **a.** Lysates from OCI-AML3 FLAG-HA-dTAG-DCAF15[Clone#10] cells treated with 250nM dTAG-V1 for indicated times analyzed by Western blot for indicated proteins. Immunoblots representative of three independent experiments.
- **b.** Differentially-expressed genes revealed by RNA-seq analysis in OCI-AML3 FLAG-HA-dTAG-DCAF15[Clone#10] cells treated with 250nM dTAG-V1 for 24h (Top) or 72h (Bottom). Shown in blue and red, respectively, are mRNAs significantly decreased or increased (log_2(Fold-Change) < -0.3 or > 0.3). n=3 biologically independent replicates, DESeq2: two-sided Wald test adjusted with Benjamini and Hochberg method for multiple comparisons, padj<0.01.
- **c.** Bubble plot of top enriched gene sets from Enrichr for the genes upregulated in OCI-AML3 FLAG-HA-dTAG-DCAF15[Clone#10] cells after 24h and 72h of 250nM dTAG-V1 treatment.
- **d.** Cas9+ MV4-11 *TP53*-WT (sgROSA26) and *TP53*-knockout (sgTP53#4, sgTP53#6) cells treated with DMSO or 20μM etoposide (ETP) for 1h or 3h and lysates analyzed by Western blot for indicated proteins. Immunoblots representative of two independent experiments. ETP treatment induces DNA damage which activates p53 response[4].
- **e.** Heatmap showing Z-score by row for genes significantly differentially-expressed upon *DCAF15*-knockout in MV4-11 *TP53*-WT cells revealed by RNA-seq analysis (n=3 biologically independent replicates, DESeq2: two-sided Wald test adjusted with Benjamini and Hochberg method for multiple comparisons, padj<0.05).
- **f.** Normalized counts proportions (normalized to sgROSA26) for genes containing p53 transcription factor binding sites and significantly upregulated upon *DCAF15*-knockout in MV4-11 cells revealed by RNA-seq (DESeq2: two-sided Wald test adjusted with Benjamini and Hochberg method for multiple comparisons, padj<0.05). Same shown for MV4-11 *TP53*-knockout (sgTP53#6) cells. Error bars mean ± SD, n=3 biologically independent replicates. Unpaired t tests (with two-stage linear step-up procedure of Benjamini, Krieger, and Yekutieli for False Discovery Rate [FDR] multiple comparisons calculation), individual q-values listed, ns=not-significant.
- **g.** Competition-based proliferation assay performed in 7 different Cas9+ AML cell lines. Plotted values are relative sgRNA+ proportions normalized to day 3 sgRNA+ over 24 days. n=3 biologically independent replicates. Dashed line indicates 50% proliferation defect passed during course of experiment. *TP53*-WT or *TP53*-Mutant status is indicated. sgROSA26, negative control.
- **h.** Same as in (g), except that sgPCNA, positive control sgRNA, was utilized. n=3 biologically independent replicates.
- Source data are provided as a Source Data file.

Supplementary Figure 3. DCAF15 interacts with the cohesin complex.

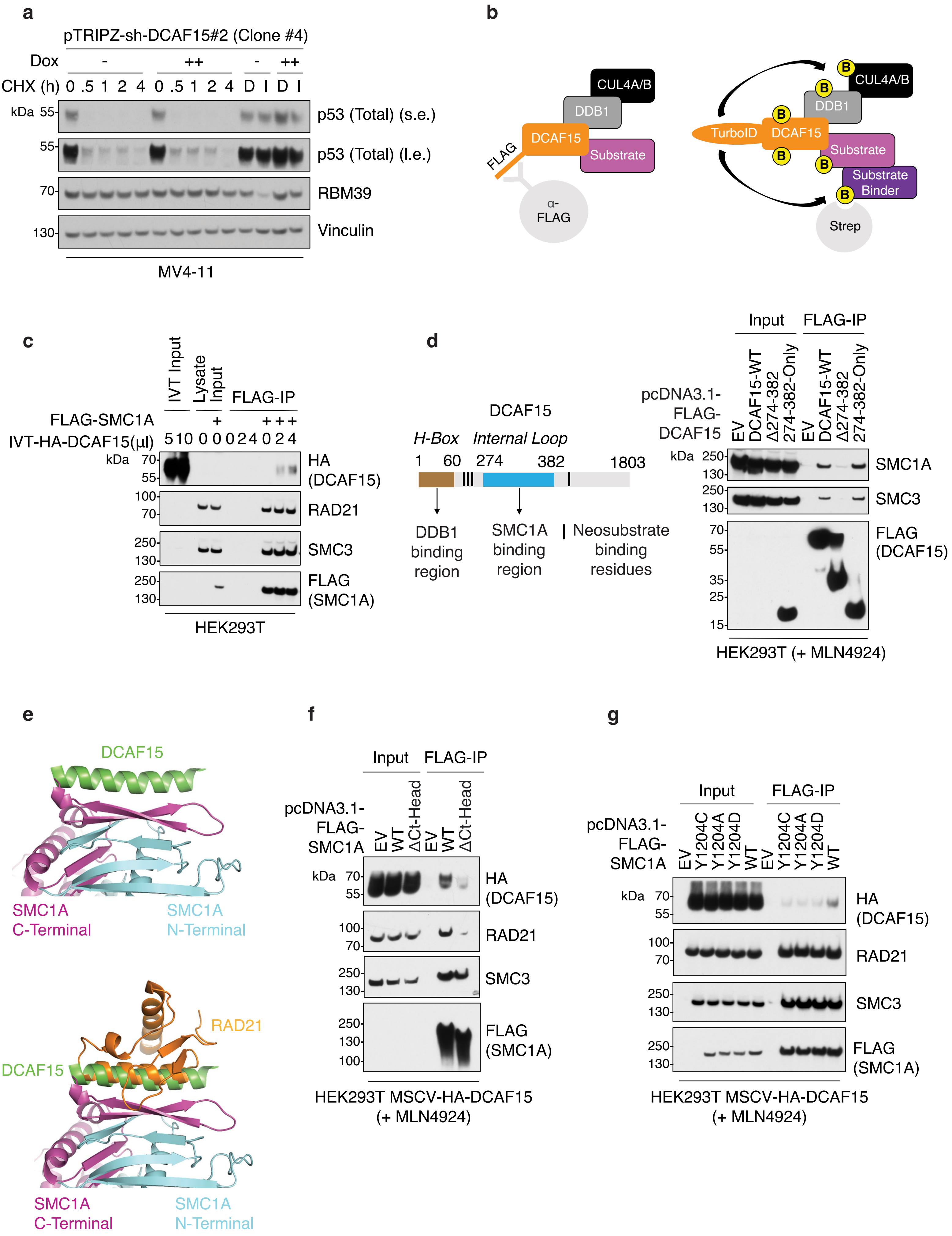

Supplementary Figure 3. DCAF15 interacts with the cohesin complex.

- **a.** Lysates from MV4-11 cells stably expressing a doxycycline (Dox)-inducible *DCAF15* shRNA plasmid and treated as indicated with Dox (1.0µg/ml for 48h; ++), cycloheximide (CHX, 20µg/ml for indicated times), DMSO (D), or indisulam (I, 3µM for 6h) analyzed by Western blot for indicated proteins. Immunoblots representative of two independent experiments.
- **b.** Left, schematic description of DCAF15 FLAG-immunoprecipitation (FLAG-IP) in HEK293T cells for identification of direct interactors by mass spectrometric analysis. α-FLAG, α-FLAG antibody agarose conjugate. Right, schematic description of DCAF15 TurboID-affinity-purification (TurboID-AP) in MV4-11 cells for identification of proximal interactors by mass spectrometric analysis. B, Biotin. Strep, Streptavidin protein magnetic-bead conjugate.
- **c.** Lysates from HEK293T cells transiently transfected with FLAG-EV or FLAG-SMC1A were subjected to FLAG-immunoprecipitation. Following, FLAG-immunoprecipitants were incubated with *in vitro* translated HA-DCAF15, and subjected to a second round of FLAG-immunoprecipitation, prior to Western blot analysis for indicated proteins. Immunoblots representative of two independent experiments.
- **d.** Left, schematic describing domains and amino acid residues of interest in DCAF15 protein. DDB1 interaction domain (H-Box, amino acids 1-60), SMC1A Internal Loop (amino acids 274-382), and molecular glue-dependent neo-substrates interaction domain (amino acids 552, 556, 559, 560, 230, 232, 235) indicated. Right, lysates from HEK293T cells transiently transfected with FLAG-EV, FLAG-DCAF15-WT, FLAG-DCAF15[∆274-382] (Internal Loop deletion mutant), or DCAF15[274-382-Only] (Internal Loop region alone), treated with 5µM MLN4924 for final 6h, subjected to FLAG-immunoprecipitation and Western blot analysis for indicated proteins. Immunoblots representative of two independent experiments.
- **e.** AlphaFold[5] prediction analysis in ChimeraX[6,7] of DCAF15 (green), SMC1A_C-Terminal (magenta), SMC1A_N-Terminal (cyan) (Top), with RAD21 (orange) superimposed (Bottom).
- **f.** Lysates from HEK293T cells stably expressing HA-tagged DCAF15 and transiently transfected with FLAG EV, FLAG-SMC1A, FLAG-SMC1A[∆Ct-Head] (C-Terminal head domain deletion mutant), treated with 5µM MLN4924 for 6h, subjected to FLAG-immunoprecipitation and Western blot analysis for indicated proteins. Immunoblots representative of three independent experiments.
- **g.** Lysates from HEK293T cells stably expressing HA-tagged DCAF15 were transiently transfected with FLAG EV, FLAG-SMC1A-WT, or FLAG-SMC1A-Point-Mutants, treated with 5µM MLN4924 for 6h, and subjected to FLAG-immunoprecipitation and Western blot analysis for the indicated proteins. Immunoblots representative of three independent experiments.
- Source data are provided as a Source Data file.

Supplementary Figure 4. DCAF15 destabilizes cohesin-bound PDS5A and CDCA5.

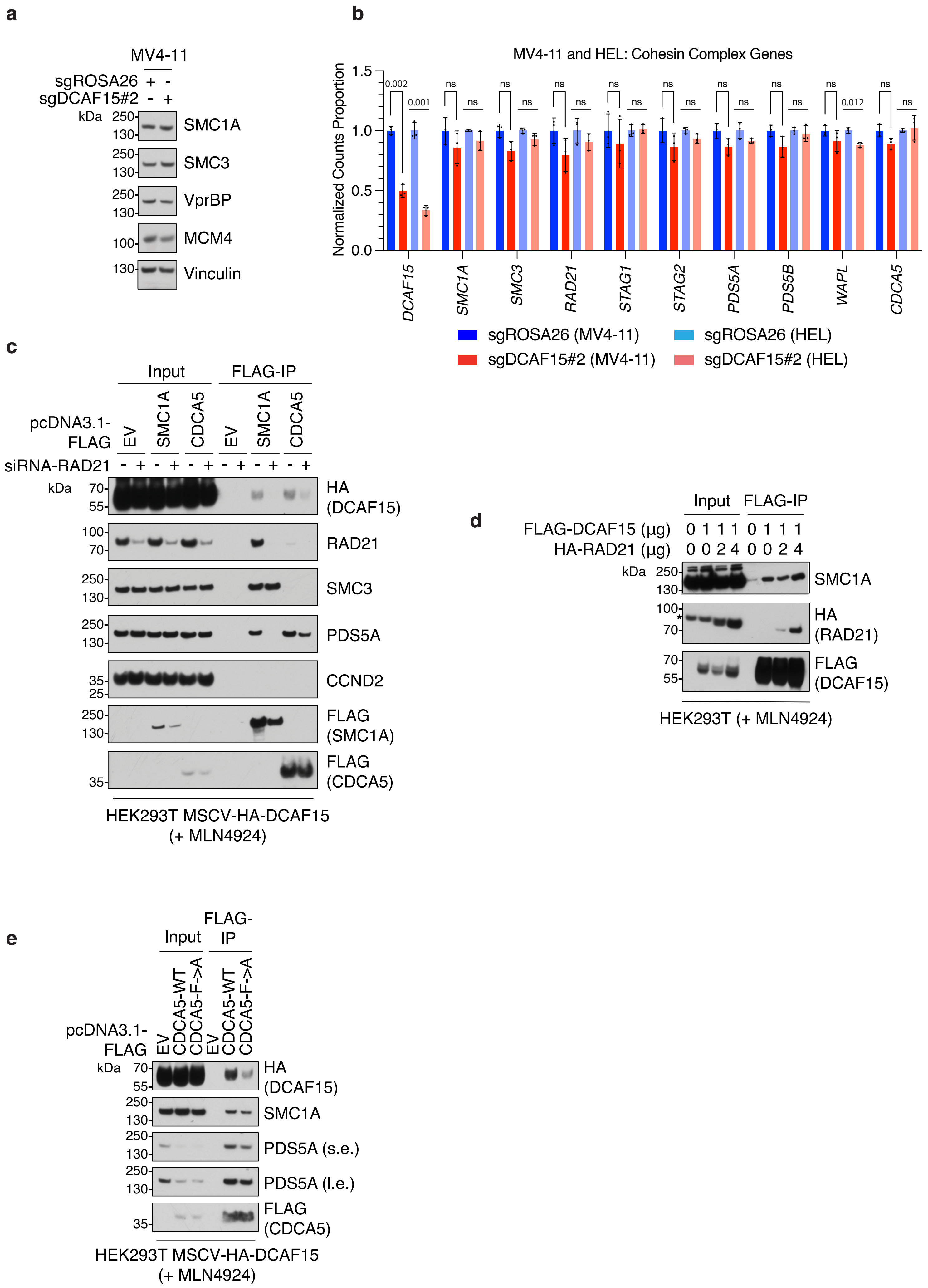

Supplementary Figure 4. DCAF15 destabilizes cohesin-bound PDS5A and CDCA5.

- **a.** Lysates from Cas9+ MV4-11 cells infected with lentiviruses encoding *ROSA26*- or *DCAF15*-targeting sgRNAs were subjected to Western blot analysis for the indicated proteins. Immunoblots are representative of three independent experiments.
- **b.** Bar graphs showing normalized counts proportions (normalized to sgROSA26) for cohesin complex genes upon *DCAF15*-knockout in MV4-11 and HEL cells revealed by RNA-seq analysis. Error bars mean ± SD, n=3 biologically independent replicates. Unpaired t tests (with two-stage linear step-up procedure of Benjamini, Krieger, and Yekutieli for False Discovery Rate [FDR] multiple comparisons calculation), individual q-values listed above graphs, ns=not-significant.
- **c.** Lysates from HEK293T cells stably expressing HA-tagged DCAF15 with the indicated siRNA and/or FLAG-tagged cDNAs, treated with 5μM MLN4924 for 6h, were subjected to FLAG-immunoprecipitation and Western blot analysis for the indicated proteins. Immunoblots are representative of two independent experiments.
- **d.** Lysates from HEK293T cells transiently transfected with FLAG-EV or FLAG-DCAF15 and an increasing titration of HA-RAD21, treated with 5μM MLN4924 for 6h, were subjected to FLAG-immunoprecipitation and Western blot analysis for the indicated proteins. Immunoblots are representative of two independent experiments. * = non-specific band.
- **e.** Lysates from HEK293T cells stably expressing HA-tagged DCAF15 were transiently transfected with FLAG-EV, FLAG CDCA5, or FLAG-CDCA5[F->A] (FGF to AGA, PDS5A-binding-motif deficient mutant[8]), treated with 5μM MLN4924 for 6h, were subjected to FLAG-immunoprecipitation and Western blot analysis for the indicated proteins. Immunoblots are representative of two independent experiments.
- Source data are provided as a Source Data file.

Supplementary Figure 5. DCAF15 sustains cohesin acetylation on chromatin.

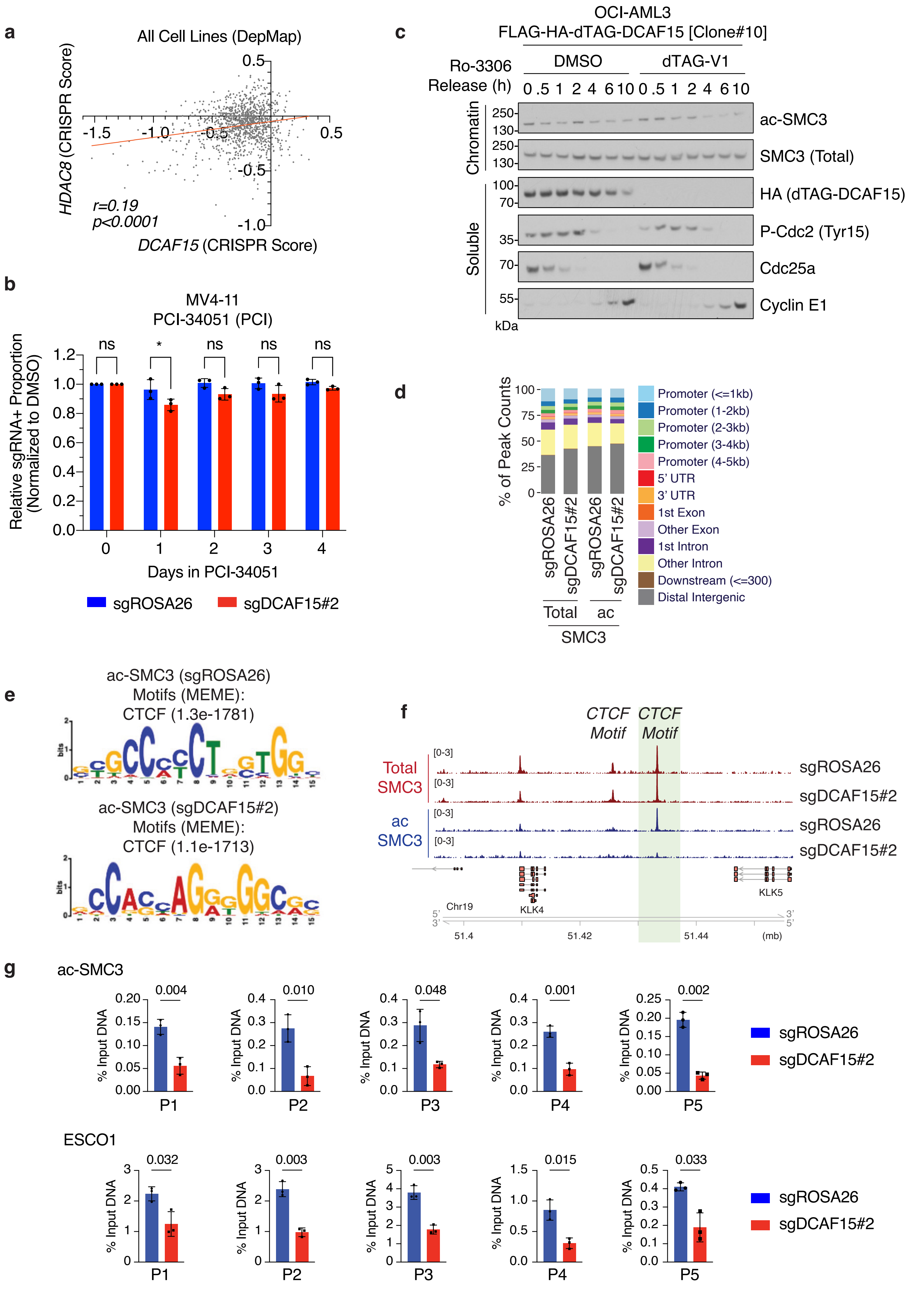

Supplementary Figure 5. DCAF15 sustains cohesin acetylation on chromatin.

- **a.** Scatter plot of *DCAF15* CRISPR Score (CS), x-axis, versus *HDAC8* CS, y-axis, for all cell lines in the Broad Institute Cancer Dependency Map (DepMap) Portal[2]. Pearson correlation, r=0.19. Simple linear regression, slope p-value<0.0001.
- **b.** Competition-based proliferation assay performed in Cas9+ MV4-11 cells infected with *ROSA26*- or *DCAF15*-targeting sgRNAs grown in presence of 10μM PCI-34051 (PCI). Plotted values are relative sgRNA+ proportion growing with PCI-34051 normalized to matched sgRNA+ proportion growing with DMSO. Error bars mean ± SD, n=3 biologically independent replicates. Two-way ANOVA (with Bonferroni's multiple comparisons test), *=padj<0.05 (padj=0.0124), ns=not-significant.
- **c.** OCI-AML3 FLAG-HA-dTAG-DCAF15[Clone#10] cells were synchronized by Ro-3306 block (5μM for 16h), and treated with DMSO or dTAG-V1 (250nM) for final 4h of block. Following, cells washed with PBS and released in fresh media containing DMSO or dTAG-V1 (250nM). Cells harvested at indicated time points, and cytoplasmic (Soluble) and nuclear (Chromatin) protein fractions analyzed by Western blot for indicated proteins. Immunoblots representative of two independent experiments.
- **d.** Distribution of significant ChIP-seq peak counts for Total-SMC3 or ac-SMC3 according to distances from transcription start sites (TSSs) and genome functional regions in Cas9+ HEL cells infected with *ROSA26*-targeting (negative control) or *DCAF15*-targeting sgRNAs. MACS2[9], p-value<$1x10^{-8}$.
- **e.** MEME-ChIP[10] analysis of ac-SMC3 peaks from Cas9+ HEL cells infected with lentiviruses encoding *ROSA26*- or *DCAF15*-targeting sgRNA plasmids.
- **f.** Representative genome browser track (produced using Integrative Genomics Viewer) showing Total-SMC3 and ac-SMC3 levels at a tandem CTCF site in Cas9+ HEL cells infected with *ROSA26* or *DCAF15*-targeting sgRNAs.
- **g.** Bar graphs showing quantification (% Input DNA) of ChIP-qPCR signals for ac-SMC3 (Top) and ESCO1 (Bottom) from Cas9+ HEL cells infected with *ROSA26*- or *DCAF15*-targeting sgRNAs. Primer pairs (P1-P5) were selected to amplify genetic loci with ESCO1 occupancy at baseline as demonstrated in Rahman *et al.*[1]. ac-SMC3 and ESCO1 ChIP-qPCR signals normalized to corresponding Total-SMC3 signal. Error bars mean ± SD, n=3 biologically independent replicates. Two-tailed unpaired t tests (with Welch's correction and two-stage linear step-up procedure of Benjamini, Krieger, and Yekutieli for False Discovery Rate [FDR] multiple comparisons calculation), individual q-values listed above graphs.
- Source data are provided as a Source Data file.

Supplementary Figure 6. *DCAF15* loss results in enlargement of chromatin loops.

a

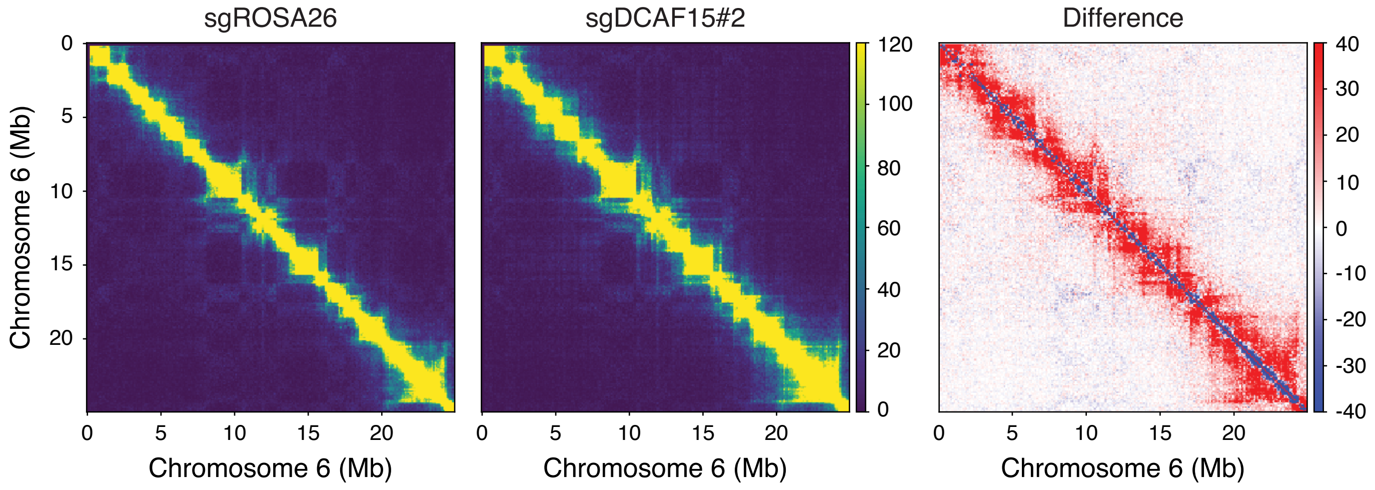

Supplementary Figure 6. *DCAF15* loss results in enlargement of chromatin loops.

- **a.** Comparison of differential normalized Hi-C contact matrices in Cas9+ HEL cells infected with lentiviruses encoding *ROSA26*-targeting (negative control) or *DCAF15*-targeting sgRNAs in a region of chromosome 6 (chr6:00,000,000-25,000,000) that exemplifies changes at TAD-scale distances. Hi-C data derived from 2 independent biological replicates in each condition.

Supplementary Figure 7. *DCAF15* loss disrupts DNA replication fork integrity.

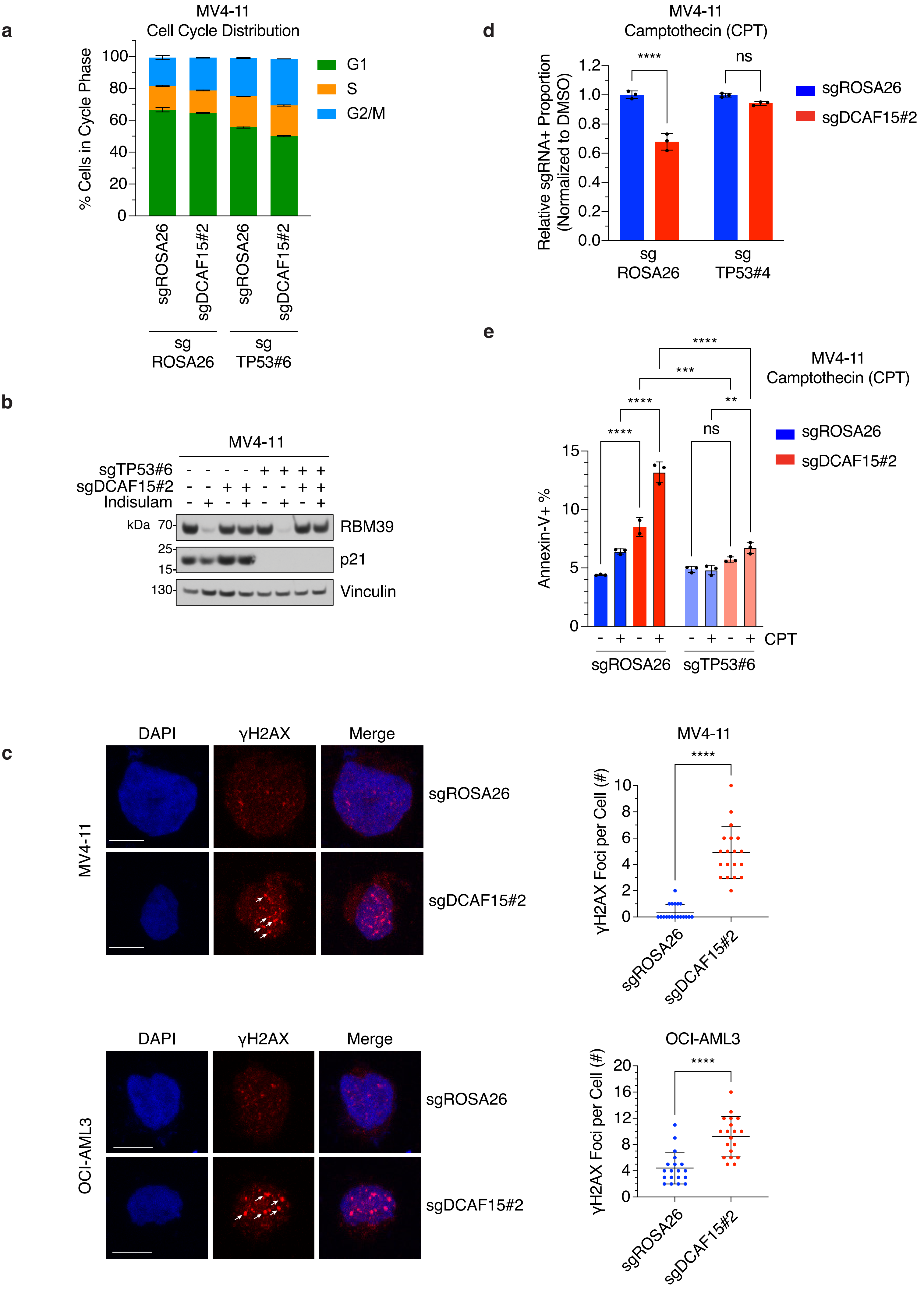

Supplementary Figure 7. *DCAF15* loss disrupts DNA replication fork integrity.

- **a.** Cas9+ MV4-11 *TP53*-WT (sgROSA26) and *TP53*-knockout (sgTP53#6) cells were infected with lentiviruses encoding *ROSA2*6- or *DCAF15*-targeting sgRNAs. 3 days post-infection, cells were stained with Hoechst 33342 solution (cell-permeable DNA dye) and subjected to flow cytometric analysis for cell cycle phase quantification based on DNA content amount. Error bars mean ± SD, n=3 biologically independent replicates.
- **b.** Cas9+ MV4-11 *TP53*-WT (sgROSA26) and *TP53*-knockout (sgTP53#6) cells were infected with lentiviruses encoding *ROSA2*6- or *DCAF15*-targeting sgRNAs and treated with DMSO or 3μM indisulam for 6h. Lysates were analyzed by Western blot for the indicated proteins. Immunoblots are representative of three independent experiments.
- **c.** Cas9+ MV4-11(Top) and OCI-AML3 (Bottom) cells infected with lentiviruses encoding *ROSA2*6- or *DCAF15*-targeting sgRNAs were adhered to coverslips and stained for immunofluorescence quantification of γH2AX DNA double-strand break damage foci. Left, representative images with arrows indicating examples of individual γH2AX foci. Scale-bars = 10μm. Right, quantification of number of γH2AX foci per cell (n=19 cells per condition). Error bars mean ± SD. Two-tailed unpaired t test with Welch's correction, ****=p-value<0.0001.
- **d.** Cas9+ MV4-11 *TP53*-WT (sgROSA26) and *TP53*-knockout (sgTP53#4) cells were infected with lentiviruses encoding *ROSA26*-targeting or *DCAF15*-targeting sgRNAs and grown in the presence of DMSO or 2nM camptothecin (CPT) for 4 days. sgRNA+ populations were assessed for mCherry expression by flow cytometry. Relative sgRNA+ proportion quantification is shown for cells growing with CPT normalized to matched sgRNA+ proportion growing with DMSO. Error bars mean ± SD, n=3 biologically independent replicates. Two-way ANOVA (with Bonferroni's multiple comparisons test), ****=padj<0.0001, ns=not-significant.
- **e.** Cas9+ MV4-11 *TP53*-WT (sgROSA26) and *TP53*-knockout (sgTP53#6) cells were infected with lentiviruses containing mCherry-co-expressed *ROSA2*6-targeting or *DCAF15*-targeting sgRNAs. On day 3 post-infection, cells were treated with DMSO or 1nM camptothecin (CPT) for 24h, and then stained with Annexin V, Pacific Blue conjugate and subjected to flow cytometric analysis for quantification of early-apoptosis induction. Error bars mean ± SD, n=3 biologically independent replicates. Two-way ANOVA (with Tukey's multiple comparisons test), ****=padj<0.0001; ***=padj<0.001 (padj=0.0002); **=padj<0.01 (padj=0.0034); ns=not-significant.
- Source data are provided as a Source Data file.

Supplementary Figure 8. Flow cytometry gating strategies.

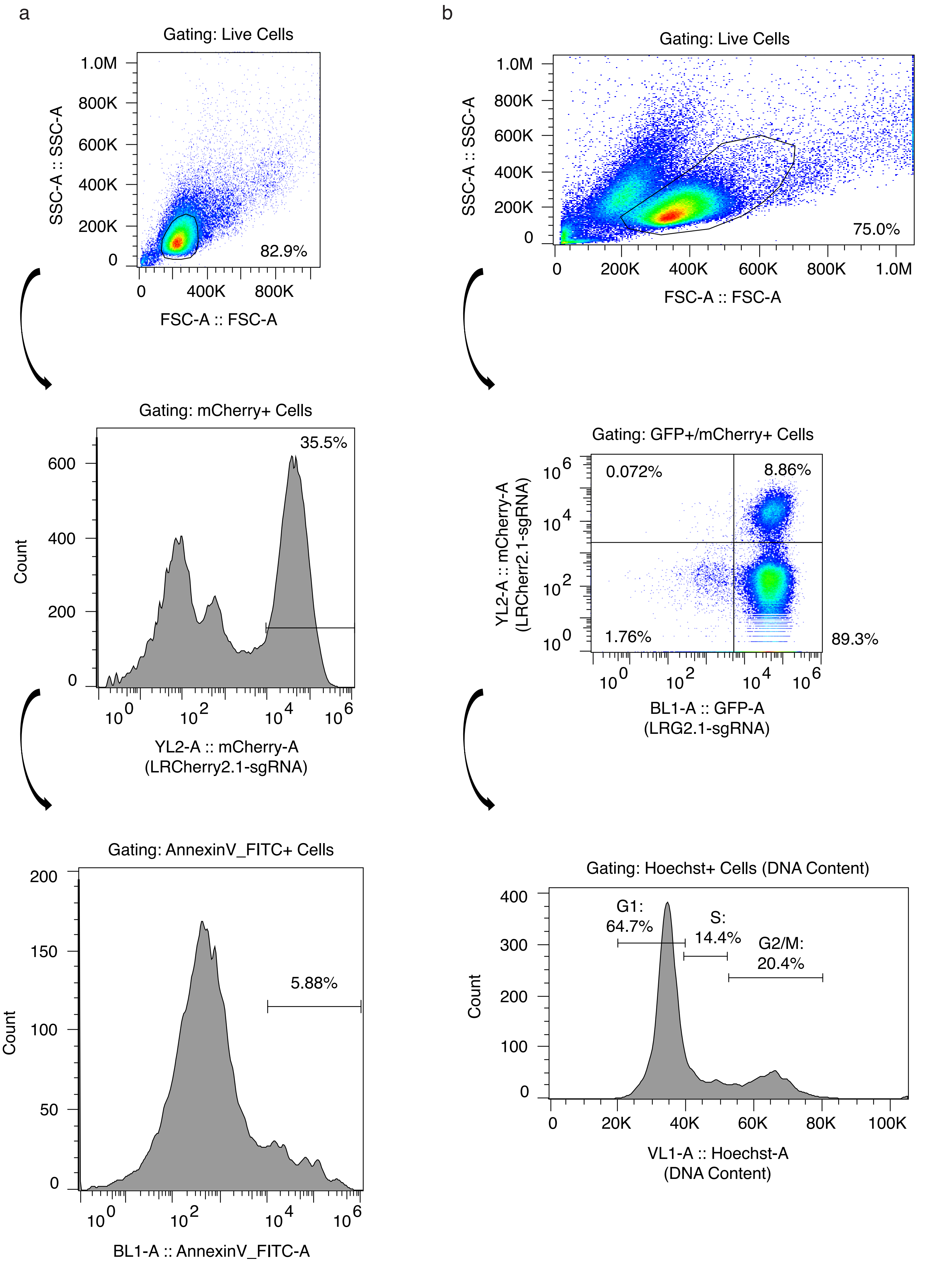

Supplementary Figure 8. Flow cytometry gating strategies.

- **a.** Example of flow cytometry gating strategy for isolating 1) live cells, followed by 2) mCherry+ cells (containing lentivirally-transduced LRCherry2.1-sgRNA), followed by 3) AnnexinV_FITC+ cells (for quantification of early-apoptosis induction). Data shown pertains to Fig. 2i.
- **b.** Example of flow cytometry gating strategy for isolating 1) live cells, followed by 2) GFP+/mCherry+ cells (containing both lentivirally-transduced LRG2.1-sgRNA and LRCherry2.1-sgRNA), followed by 3) Hoechst+ cells (for quantification of DNA content, and determination of cell cycle phase distribution). Data shown pertains to Supplementary Fig. 7a.
