# Supplementary material for: DCAF15 control of cohesin dynamics sustains acute myeloid leukemia
Source: Nat Commun. 2024 Jul 3;15:5604. doi: 10.1038/s41467-024-49882-x (PMC11222469; doi:10.1038/s41467-024-49882-x)
Supplement: Supplementary file 3 — Description of Additional Supplementary Files [file 41467_2024_49882_MOESM3_ESM.pdf]

Description of Additional Supplementary Files

File Name: Supplementary Data 1
Description: **CRISPR sgRNA sequences and CRISPR screen data.** Sequences of CRISPR sgRNAs used, and raw data from CRISPR screens.

File Name: Supplementary Data 2
Description: **RNA-seq data.** Raw data from RNA-seq analyses. n=3 biologically independent replicates; DESeq2: two-sided Wald test adjusted with Benjamini and Hochberg method for multiple comparisons.

File Name: Supplementary Data 3
Description: **Mass spec data.** Raw data from mass spec analyses.

File Name: Supplementary Data 4
Description: **ChIP-seq data.** Raw data from ChIP-seq analyses.
