# Supplementary material for: DCAF15 control of cohesin dynamics sustains acute myeloid leukemia
Source: Nat Commun. 2024 Jul 3;15:5604. doi: 10.1038/s41467-024-49882-x (PMC11222469; doi:10.1038/s41467-024-49882-x)
Supplement: Supplementary file 8 — Reporting Summary [file 41467_2024_49882_MOESM8_ESM.pdf]

nature portfolio

Corresponding author(s): Luca Busino

Last updated by author(s): Jun 14, 2024

Reporting Summary

Nature Portfolio wishes to improve the reproducibility of the work that we publish. This form provides structure for consistency and transparency in reporting. For further information on Nature Portfolio policies, see our Editorial Policies and the Editorial Policy Checklist.

Statistics

For all statistical analyses, confirm that the following items are present in the figure legend, table legend, main text, or Methods section.

n/a	Confirmed	
☐	☒	The exact sample size (*n*) for each experimental group/condition, given as a discrete number and unit of measurement
☐	☒	A statement on whether measurements were taken from distinct samples or whether the same sample was measured repeatedly
☐	☒	The statistical test(s) used AND whether they are one- or two-sided<br>*Only common tests should be described solely by name; describe more complex techniques in the Methods section.*
☒	☐	A description of all covariates tested
☐	☒	A description of any assumptions or corrections, such as tests of normality and adjustment for multiple comparisons
☐	☒	A full description of the statistical parameters including central tendency (e.g. means) or other basic estimates (e.g. regression coefficient) AND variation (e.g. standard deviation) or associated estimates of uncertainty (e.g. confidence intervals)
☐	☒	For null hypothesis testing, the test statistic (e.g. *F*, *t*, *r*) with confidence intervals, effect sizes, degrees of freedom and *P* value noted<br>*Give P values as exact values whenever suitable.*
☒	☐	For Bayesian analysis, information on the choice of priors and Markov chain Monte Carlo settings
☒	☐	For hierarchical and complex designs, identification of the appropriate level for tests and full reporting of outcomes
☒	☐	Estimates of effect sizes (e.g. Cohen's *d*, Pearson's *r*), indicating how they were calculated

Our web collection on statistics for biologists contains articles on many of the points above.

Software and code

Policy information about availability of computer code

Data collection	No custom or commercial code was used to collect data.
Data analysis	The following software was used in our study:<br>Bowtie 2 (Bowtie 2 (v2.4.5) with default parameters was used for alignment)<br>SAMtools (SAMtools (v1.15.1) was used for converting sam file to bam file, sorting and removing duplicates)<br>MACS2 (v2.2.9.1) (MACS2 was used for peak calling with the parameters “--nomodel -p 1E-10 --keep-dup all”)<br>MEME-ChIP (v5.5.5)<br>FastQC (v0.11.9) was used for ChIP-seq data quality control, and low quality parts of the reads were trimmed by cutadapt (v4.1)<br>deepTools (v3.5.1) module bamCoverage was used for converting BAM files to bigWig files<br>featureCounts (v2.0.2) was used for calculating the ChIP-seq signal of specific regions<br>UCSC Genome Browser (University of California Santa Cruz Genomics Institute)<br>Integrative Genomics Viewer (IGV) (v2.16.0)<br>STAR RNA-seq Aligner (v2.7.11a)<br>R Bioconductor DESeq2 Package (v1.44.0)<br>Juicer (v1.6)<br>HiCExplorer (v3.7.2)<br>coolpup.py (v1.0.0)<br>MaxQuant (v1.6.17.0)<br>AlphaFold (v2.3.2)<br>UCSF ChimeraX (v1.6)<br>Fiji Software (ImageJ2 v2.14.0)

FlowJo Software (v10.4)
Prism 10 (v10.2.3) Software (Graphpad by Dotmatics) was used for all statistical analyses

For manuscripts utilizing custom algorithms or software that are central to the research but not yet described in published literature, software must be made available to editors and reviewers. We strongly encourage code deposition in a community repository (e.g. GitHub). See the Nature Portfolio guidelines for submitting code & software for further information.

Data

Policy information about availability of data

All manuscripts must include a data availability statement. This statement should provide the following information, where applicable:
- Accession codes, unique identifiers, or web links for publicly available datasets
- A description of any restrictions on data availability
- For clinical datasets or third party data, please ensure that the statement adheres to our policy

Next generation sequencing data are deposited in the Gene Expression Omnibus (GEO) database with the accession number GSE241581 [https://www.ncbi.nlm.nih.gov/geo/query/acc.cgi?acc=GSE241581].

Mass spectrometry proteomics data are deposited in the MassIVE and ProteomeXchange data repositories with the accession numbers MSV000092697 [https://massive.ucsd.edu/ProteoSAFe/dataset.jsp?task=fde90091ea2c4f4c86f69299de2f830e] and PXD044661 [https://proteomecentral.proteomexchange.org/cgi/GetDataset?ID=PXD044661], respectively.

There are no restrictions on these data. Source data are provided with this paper.

Research involving human participants, their data, or biological material

Policy information about studies with human participants or human data. See also policy information about sex, gender (identity/presentation), and sexual orientation and race, ethnicity and racism.

Reporting on sex and gender	Our study did not use human research participants.
Reporting on race, ethnicity, or other socially relevant groupings	Our study did not use human research participants.
Population characteristics	Our study did not use human research participants.
Recruitment	Our study did not use human research participants.
Ethics oversight	Our study did not use human research participants.

Note that full information on the approval of the study protocol must also be provided in the manuscript.

Field-specific reporting

Please select the one below that is the best fit for your research. If you are not sure, read the appropriate sections before making your selection.

☒ Life sciences ☐ Behavioural & social sciences ☐ Ecological, evolutionary & environmental sciences

For a reference copy of the document with all sections, see nature.com/documents/nr-reporting-summary-flat.pdf

Life sciences study design

All studies must disclose on these points even when the disclosure is negative.

Sample size	No statistical method was used to predetermine sample size. For quantified experiments, n=3 was used, as a minimum.
Data exclusions	Data was not excluded.
Replication	Experiments described were repeated on at least two separate occasions with similar results as those represented in the figures. Statistics and Reproducibility information can be found in the Figures Legends and/or Methods section.
Randomization	Randomization was not required for in vitro studies given that proper controls were included, so all cell lines would receive the same treatment, regardless of subtype.
Blinding	Investigators were blinded to sample identity and group for DNA fiber assay analysis. For other analyses, authors were not blinded since no subjective analysis was performed that would be biased by the investigator knowing the sample identity. Objective data analysis was measured using software listed above.

Reporting for specific materials, systems and methods

We require information from authors about some types of materials, experimental systems and methods used in many studies. Here, indicate whether each material, system or method listed is relevant to your study. If you are not sure if a list item applies to your research, read the appropriate section before selecting a response.

Materials & experimental systems

n/a	Involved in the study
☐	☒ Antibodies
☐	☒ Eukaryotic cell lines
☒	☐ Palaeontology and archaeology
☒	☐ Animals and other organisms
☒	☐ Clinical data
☒	☐ Dual use research of concern
☒	☐ Plants

Methods

n/a	Involved in the study
☐	☒ ChIP-seq
☐	☒ Flow cytometry
☒	☐ MRI-based neuroimaging

Antibodies

Antibodies used

Primary antibodies used for Western blotting in this paper and/or the Supplementary Information include the following:
CAPER (“RBM39”) (Bethyl Laboratories, A300-291A-T, 1:2,000)
HA-Tag (Cell Signaling Technology, 3724S, 1:2,000)
Vinculin (Santa Cruz Biotechnology, sc-73614, 1:8,000)
p21 (Santa Cruz Biotechnology, sc-6246, 1:)250
p53 (Bethyl Laboratories, A300-247A-T, 1:1,000)
Cleaved Caspase-3 (Asp175) (Cell Signaling Technology, 9661T, 1:500)
SMC1 (“SMC1A”) (Bethyl Laboratories, A300-055A-T, 1:1,000)
SMC3 (Bethyl Laboratories, A300-060A-T, 1:1,000)
RAD21 (Bethyl Laboratories, A300-080A-T, 1:1,000)
SA2 (“STAG2”) (Bethyl Laboratories, A302-580A-T, 1:1,000)
SCC-122 (“PDS5A”) (Bethyl Laboratories, A300-089A-T, 1:1,000)
CDCA5 (“Sororin”) (Abcam, ab192237, 1:)500
Cyclin D2 (Cell Signaling Technology, 3741T, 1:1,000)
DDB1 (Bethyl Laboratories, A300-462A-T, 1:1,000)
FLAG-Tag (Millipore Sigma, F7425, 1:6,000)
WAPL (Proteintech, 16370-1-AP, 1:500)
SA1 (“STAG1”) (Bethyl Laboratories, Cat#A302-579A-T, 1:2,000)
PDS5B (Bethyl Laboratories, A300-538A-T, 1:2,000)
Ubiquitin (K48) (Cell Signaling Technology, 8081S, 1:1,000)
Acetyl-SMC3 (Lys105/106) (Millipore Sigma, MABE1073, 1:1,000)
HDAC8 (Proteintech, 17548-1-AP, 1:500)
Phospho-Cdc2 (Tyr15) (Santa Cruz Biotechnology, sc-136014, 1:250)
Cdc25A (Santa Cruz Biotechnology, sc-7389, 1:250)
Phospho-Histone-H3 (Ser10) (Millipore Sigma, 06-570, 1:1,000)
Gamma-H2AX (Bethyl Laboratories, A300-081A-T, 1:500)
VprBP (Bethyl Laboratories, A301-887A, 1:1,000)
MCM4 (Proteintech, 13043-1-AP, 1:1,000)
Cyclin E1 (Cell Signaling Technology, 4129T, 1:1,000)

Secondary antibodies used for Western blotting in this paper and/or the Supplementary Information include the following:
Goat anti-rabbit IgG, HRP-linked (Cell Signaling Technology, 7074S, 1:10,000)
Sheep anti-mouse IgG, HRP-linked (Cytiva, NA931-1ML, 1:10,000)

Primary antibody used for endogenous-SMC1A-IP includes the following:
SMC1A (Abcam, ab140493, 1-10µg per IP)

Primary antibodies used for ChIP-seq include the following:
SMC3 (Bethyl Laboratories, A300-060A, 12µg per IP)
Acetyl-SMC3 (Lys105/106) (EMD Millipore, MABE1073, 20µg per IP)

Primary antibody used for ChIP-qPCR:
ESCO1 (gift from Susannah Rankin, Oklahoma Medical Research Foundation, 20µg per IP)

Primary antibodies used for flow cytometry-based apoptosis-induction assay include the following:
Annexin V, Pacific Blue conjugate (Thermo Fisher Scientific, A35122, 2.5µl in 50µl cell suspension)
Annexin V, FITC conjugate (Thermo Fisher Scientific, A13199, 2.5µl in 50µl cell suspension)

Primary antibodies used for DNA fiber assay include the following:
CldU (Abcam, ab6326, 1:200)

	IdU (BD Pharmigen, 347580, 1:40)<br><br>Secondary antibodies used for DNA fiber assay include the following:<br>Goat anti-rat IgG, Alexa Fluor 647 conjugate (Thermo Fisher Scientific, A-21247, 1:100)<br>Goat anti-mouse IgG, Alexa Fluor 488 conjugate (Thermo Fisher Scientific, A-11001, 1:100)
Validation	All antibodies were used in accordance to the manufacturer guidelines and have been well documented in the literature. When possible, antibodies were validated beyond the manufacturers data sheet specification by staining blots (Western) with known deficiencies for expression of the antigen.

Eukaryotic cell lines

Policy information about cell lines and Sex and Gender in Research

Cell line source(s)	Cell lines used in this study include (all human):<br>MOLM-13 (DSMZ, ACC-554)<br>MV4-11 (ATCC, CRL-9591)<br>THP-1 (ATCC, TIB-202)<br>HEL (ATCC, TIB-180)<br>OCI-AML3 (DSMZ, ACC-582)<br>SET-2 (DSMZ, ACC-608)<br>U-937 (ATCC, CRL-1593.2)<br>Jurkat (ATCC, TIB-152)<br>U-2932 (DSMZ, ACC-633)<br>OPM-1 (DSMZ, ACC-50)<br>Hep-G2 (ATCC, HB-8065)<br>HEK-293 (ATCC, CRL-1573)<br>HEK-293T (ATCC, CRL-3216)
Authentication	We did not conduct cell line authentication of these commonly-used cell lines beyond that of the manufacturers.
Mycoplasma contamination	Cell lines were tested negative by the cell line manufacturers.
Commonly misidentified lines<br>(See ICLAC register)	We have not utilized any commonly misidentified cell lines in our study.

Plants

Seed stocks	Our study did not use plants.
Novel plant genotypes	Our study did not use plants.
Authentication	Our study did not use plants.

ChIP-seq

Data deposition

☒ Confirm that both raw and final processed data have been deposited in a public database such as GEO.

☒ Confirm that you have deposited or provided access to graph files (e.g. BED files) for the called peaks.

Data access links<br>*May remain private before publication.*	ChIP-seq data associated with this study has been deposited into the Gene Expression Omnibus (GEO) data repository with the accession number GSE241581.
Files in database submission	The following files are available in the database submission:<br>HEL cells, LRCherry2.1-sgROSA26(Control), Input<br>HEL cells, LRCherry2.1-sgROSA26(Control), total-SMC3, ChIP<br>HEL cells, LRCherry2.1-sgROSA26(Control), acetyl-SMC3, ChIP<br>HEL cells, LRCherry2.1-sgDCAF15#2, total-SMC3, ChIP<br>HEL cells, LRCherry2.1-sgDCAF15#2, acetyl-SMC3, ChIP
Genome browser session<br>(e.g. UCSC)	No longer applicable.

Methodology

Replicates	Each ChIP-seq experimental condition used had n=1 biological replicate.
Sequencing depth	The total number of reads for the total-SMC3 data are ~17,000,000 and ~25,000,000.<br>The total number of reads for the acetyl-SMC3 data are ~23,000,000 and ~19,000,000.<br>The total number of reads for the input data is ~26,000,000.

	The length of reads for all the sequencing data is 76bp and they are single-end sequencing data.
Antibodies	Primary antibodies used for ChIP-seq include the following:<br>SMC3 (Bethyl Laboratories, A300-060A)<br>acetyl-SMC3 (Lys105/106) (EMD Millipore, MABE1073)
Peak calling parameters	FASTQ files were aligned with Bowtie2, reads were sorted and duplicates removed with SAMtools, and peaks were called with MACS2.
Data quality	FastQC (v0.11.9) was used for data quality control, and low quality parts of the reads were trimmed by cutadapt (v4.1). All the peaks meet the criteria of FDR < 5%.
Software	Bowtie2 (v2.4.5) with default parameters was used for alignment. SAMtools (v1.15.1) was used for converting SAM files to BAM files, sorting and removing duplicates. MACS2 was used for peak calling with the parameters “--nomodel -p 1E-10 --keep-dup all”. deepTools (v3.5.1) module bamCoverage was used for converting BAM files to bigWig files. featureCounts (v2.0.2) was used for calculating the ChIP-seq signal of specific regions, and RPM was used for ChIP-seq signal normalization.

Flow Cytometry

Plots

Confirm that:

☐ The axis labels state the marker and fluorochrome used (e.g. CD4-FITC).

☐ The axis scales are clearly visible. Include numbers along axes only for bottom left plot of group (a 'group' is an analysis of identical markers).

☐ All plots are contour plots with outliers or pseudocolor plots.

☒ A numerical value for number of cells or percentage (with statistics) is provided.

Methodology

Sample preparation	Live and dead cells were harvested and single cell suspensions were prepared and resuspended in FACS buffer containing 10% serum.
Instrument	The Thermo Fisher Scientific Attune NxT Flow Cytometer was used for in vitro experiments.
Software	Flow cytometry data was analyzed using FlowJo Software.
Cell population abundance	Reported as the percentage of total events after gating out debris.
Gating strategy	Single live cells were determined by gating out debris and clumps using FSC/SSC.<br><br>Distinct right-shifted histogram peaks in relevant Thermo Fisher Scientific Attune NxT Flow Cytometer channels were gated as GFP+ (Channel: BL1) or mCherry+ (Channel: YL2) fluorescent populations, in order to track cells lentivirally-infected with LRG2.1 (GFP+) or mCherry2.1 (mCherry+) sgRNA vectors.<br><br>AnnexinV-PacificBlue_HIGH (Channel: VL1) or AnnexinV-FITC_HIGH (Channel: BL1) fluorescent populations were gated as AnnexinV+ cells.<br><br>Cellular DNA content was assessed by live cell staining and quantification with Hoechst (Channel: VL1).

☒ Tick this box to confirm that a figure exemplifying the gating strategy is provided in the Supplementary Information.
